# Adipose tissue inflammation linked to obesity: A review of current understanding, therapies and relevance of phyto-therapeutics

**DOI:** 10.1016/j.heliyon.2023.e23114

**Published:** 2023-12-02

**Authors:** Christiana Eleojo Aruwa, Saheed Sabiu

**Affiliations:** Department of Biotechnology and Food Science, Durban University of Technology, PO Box 1334, Durban, 4000, South Africa

**Keywords:** Adipose tissue inflammation, Obesity, Comorbidity, Phytotherapy, Phenols, Macromolecular antioxidants

## Abstract

Obesity is a current global challenge affecting all ages and is characterized by the up-regulated secretion of bioactive factors/pathways which result in adipose tissue inflammation (ATI). Current obesity therapies are mainly focused on lifestyle (diet/nutrition) changes. This is because many chemosynthetic anti-obesogenic medications cause adverse effects like diarrhoea, dyspepsia, and faecal incontinence, among others. As such, it is necessary to appraise the efficacies and mechanisms of action of safer, natural alternatives like plant-sourced compounds, extracts [extractable phenol (EP) and macromolecular antioxidant (MA) extracts], and anti-inflammatory peptides, among others, with a view to providing a unique approach to obesity care. These natural alternatives may constitute potent therapies for ATI linked to obesity. The potential of MA compounds (analysed for the first time in this review) and extracts in ATI and obesity management is elucidated upon, while also highlighting research gaps and future prospects. Furthermore, immune cells, signalling pathways, genes, and adipocyte cytokines play key roles in ATI responses and are targeted in certain therapies. As a result, this review gives an in-depth appraisal of ATI linked to obesity, its causes, mechanisms, and effects of past, present, and future therapies for reversal and alleviation of ATI. Achieving a significant decrease in morbidity and mortality rates attributed to ATI linked to obesity and related comorbidities is possible as research improves our understanding over time.

## Abbreviations

ACE: Angiotensin converting enzyme
AIHs: Anti-inflammatory hormones
AINs: Anti-inflammatory neuropeptides
AIPs: Anti-inflammatory peptides
ApoC3: Apolipoprotein C3
ASPCs: Adipose stem and progenitor cells
AT: Adipose tissue
ATI: Adipose tissue inflammation
ATIR: Adipose tissue related insulin resistance
ATMs: Adipose tissue macrophages
BAPs: Bioactive peptides
BeAT: Beige adipose tissue
BMI: Body mass index
BrAT: Brown adipose tissue
COX-2: Cyclooxygenase-2
CVD: Cardiovascular disease
DHA: Docosahexaenoic
DPP4: Dipeptidyl peptidase 4
EA: Electroacupuncture
ECM: Extracellular matrix
EP: Extractable phenol (EP)
EPA: Eicosapentaenoic acid
EPO: Endogenous Erythropoietin
ERK: Extracellular signal-regulated kinase
GABA: γ-aminobutyric acid
GLP-1: Glucagon-like peptide 1
H^+^: Hydrogen ion
HDL: High density lipoprotein
HMG-CoA reductase: 3-hydroxy-3-methlyglutaryl coenzyme A
HNF1α: Hepatocyte nuclear factor-1 alpha
IL-6: Interleukin 6 (IL-6)
ILC2s: Innate Lymphoid type 2 cytokines
IR: Insulin resistance
IRS1: Insulin receptor substarte-1
JNK: c-Jun N-terminal kinase
LDL: Low-density lipoprotein
LPS: Lipopolysaccharide
MA: Macromolecular antioxidant
MAPK: Mitogen-activated protein kinase
MC: Melanocortin
MC4R: Melanocortin-4 receptor
MCP-1: Monocyte chemotactic protein 1
mTOR: Mammalian target of rapamycin
NCD: Non-communicable disease
NEPA: Non-extractable proanthocyanidins
NEPPs: Non-extractable polyphenols
NF-κB: Nuclear factor-κB
NPC1L1: Niemann-Pick C1-Like 1
Nrf2: Nuclear factor erythroid 2–related factor 2
OH: Hydroxyl radical
OS: Oxidative stress
PAI-1: Plasminogen activator inhibitor-1
PGC-1α: Pparg coactivator 1 alpha
PI3K: Phosphatidylinositol 3-kinase
PPAR: Peroxisome proliferator-activated receptors
PUFA: Polyunsaturated fatty acid
SREBP2: Sterol regulatory element-binding protein 2
T2D: Type 2 diabetes
TCM: Traditional Chinese Medicine
TFAM: Mitochondrial transcription factor A
TFs: Transcription factors
TGs: Triglycerides
TNF-a: Tumour necrosis factor
TPX: 1,3,6,7-Tetrahydroxy-8-prenylxanthone
USFDA: United States Department of Food and Drug Administration
VEGFA: Vascular endothelial growth factor A
VLDL: Very-low-density lipoprotein
WAT: White adipose tissue
WATI: White adipose tissue inflammation

## 1 Introduction

Obesity is a global epidemic affecting all age-groups. The disease has shown a significant increase in incidence in the past decade, especially in developed nations [1,2]. It is a multifaceted, non-communicable disease (NCD) that leads to health complications. Obesity comorbidities include type 2 diabetes (T2D) and insulin resistance (IR) [3], hypertension, cardiovascular disease (CVD), cancer, liver disease, obstructive sleep apnoea, among others. These obesity complications account for the high morbidity and mortality rates in obese individuals [4]. A combination of cancer (e.g., pancreatic ductal adenocarcinoma linked to visceral adiposity) [5], diabetes [6], and atherosclerosis linked to obesity was predicted to significantly reduce average life expectancy in 2020 [7]. As a result, part of the WHO's 2030 sustainable development goal is targeted at reducing NCD-related deaths by up to a third through treatment and preventive options [7]. Surprisingly, childhood obesity is also on the rise in developing nations where 1 in 3 children below the age of 5 are either obese or undernourished. Globally, about 340 million adolescents (ages 5–19) have also been reported to be obese. This constitutes an increase of 8.1 % between the year 2000 and 2018 [8]. The upward regulation of ageing cells in adipose organs is also linked to obesity onset, and aged cells contribute to increase levels of inflammatory factors [[9], [10], [11]]. The development of an obese state is linked to fats build up in adipose tissue (AT) depots [12]. Furthermore, in spite of pharmaceutical advancements, obesity and its comorbidities hardly have cures and are mostly managed conditions [13,14].

In terms of comorbidities, as part of the aetiology of IR development, innate/adaptive immunity plays a key role [15]. Studies have demonstrated that adaptive or innate immunity abnormalities such as B and NK cells impaired function, altered macrophage polarization and T cells proliferation, occur in obesity and T2D patients [16]. Likewise, during ATI similar immune system abnormalities have been reported [17]. Also, obese, or diabetic patients often experience metabolic changes that adversely impact on adaptive/innate immunity components, their function and differentiation. For instance, leptin induces heightened Th1/Th17 cytokine and T cells generation thereby causing anti-apoptosis after antigens are stimulated in the mammalian target of rapamycin (mTOR) signalling pathway [18]. In addition, macrophages are integral in the removal of hazardous, dead, or dying (apoptotic) cells or debris from tissues. In the case of apoptotic adipocytes, their removal by AT macrophages results in ATI and obesity-linked metabolic disorders. The AT macrophage immune function is governed by a complex network of signalling molecules (polyamines, lactate, arginine, creatine) produced by adipocytes which could also have promise for stimulating or mitigating ATI [19]. As such, understanding the innate or adaptive immunity link to obesity, T2D and anti-apoptotic events could be central in the design and discovery of novel immunity-targeted therapies for ATI and IR modulation [16].

Again, since the cause of obesity is a multifactorial and not only biochemical process, an outlook on a wide range of medical options is needed to curb increasing trends. For instance, therapeutic peptides have gained scientific interest as medical alternatives for managing inflammation-linked conditions. Recent efforts have identified PEPITEM (immunopeptide) for regulation of chronic inflammation in the adiponectin-PEPITEM pathway. The pathway reduced the probability of developing T2D, and fatty liver associated with obesity by enhancing leukocyte access into ATs and inducing reduced inflammatory responses in T2D and other obesity associated disorders (hepatic steatosis). Its use in mouse models showed the peptide's ability to reverse the adverse impacts of a diet that is high in fat by increasing white blood cells and insulin synthesis in pancreatic visceral AT [20]. Biologically active peptides derived from other natural, endogenous sources have also shown anti-obesogenic effects [21]. These include milk sourced isracidin, casecidin, lactoferricin B and lactoferrin, β-casein, casoxins A-C, serorphine [22], as well as glutamate and γ-aminobutyric acid (GABA) [23]. Likewise, new anti-inflammatory peptide (AIP) analogues such as anti-obesogenic vaccine and lipase inhibitors of lipase have also been reported [24]. In additional efforts, computational studies have also been used to predict the presence of and identify AIPs. For example, unlike the *in silico* AlPpred [25] and AntiInflam [26] predictors which were based on primary protein sequence attributes, the predictor of anti-inflammatory peptide (PreAIP) integrated physicochemical, evolutionary, structural, and primary peptide features [27]. Furthermore, therapeutic peptides modelled using machine learning and genetic algorithms for cancer-related co-morbidity like iACP-GAEnsC, cACP-DeepGram [28,29], and pAtbP-EnC and iAtbP-Hyb-EnC for tuberculosis [30,31] may also hold potential as modulators of obesity and associated comorbidities.

In line with the foregoing, and to increase the emergence of synthetic and natural management or therapeutic options for ATI linked to obesity, this review aims to report on the available range of therapy options; phenolic and non-phenolic molecules, AIPs, synthetic compounds, plant-derived extracts’ [extractable phenol (EP) and macromolecular antioxidant (MA)] potentials, among other approaches (weight reduction, electroacupuncture), and their mechanisms of action and efficacies in mitigating AT inflammatory responses and pathways associated with obesity.

## 2 Obesity, adipokines and adipose tissue inflammation (ATI)

**Fig. 1:**
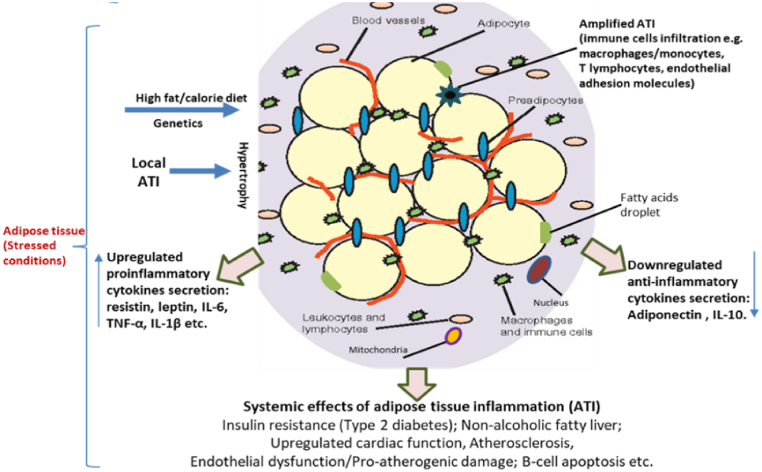
Mechanisms involved in ATI (an interplay between adipocytes and immune cells).

More than five hundred biologically active factors, for examples, adiponectin, interleukin 6 (IL-6), visfatin, resistin, tumour necrosis factor (TNF-a), are produced by the AT. Adipokines are bioactive molecules that modulate the secretion of anti-inflammatory and pro-inflammatory factors. As a major factor, adipokines function in insulin secretion and sensitivity (leptin and adiponectin), fat distribution (chemerin), homeostasis, energy expenditure, blood pressure and inflammation [progranulin, IL-6, monocyte chemotactic protein 1 (MCP-1), TNF-a] control (Fig. 1) [4]. Adipokines regulate migration of immune cells (macrophages, CD8^+^, CD4^+^ and CD3^+^ T cells) into AT and adipogenesis and have systemic effects on other target organs (heart, brain, skin, liver etc.) in the body [4,32]. In other words, up-regulation in adipokine factor synthesis could lead to functional and metabolic syndromes like hypertension, T2D. For example, if an AT is unable to store a healthy amount of fats, misplacement of fat deposits in liver and other body cell types, and inflammation of visceral AT may occur [33]. These then cause simultaneous or sequential hypoxic condition in AT, adipocyte hypertrophy, AT stress from adverse fat deposition, autophagy, and AT inflammation (ATI) (Fig. 1) [34,35].

The mechanism behind ATI development involves stressed AT (from excess, adverse fat deposits) functioning beyond their usual capacities in the liver and other body cell types, which eventually results in ATI and related systemic effects.

In the adipose tissue (AT) there are stromal-vascular and adipocyte fractions which contain varying cell types, and are of the beige (BeAT), brown (BrAT) and white (WAT) types [6]. The latter is majorly endocrine and functions in lipid storage, bioactive molecules, and hormone production [36]. In the body system, the onset of obesity has been linked to the accumulation of various immune cell types and macrophages in AT fat pads [37]. As these cells accumulate there is the development of a chronic inflammation known as ATI which contributes to IR and disrupts adipocyte cell and tissue functions [3,38]. The initiation of inflammatory pathways and up-regulated cytokines like IL-1β, IL-6 and TNF-α [36,39] also contribute to obesity and IR development. This is because ATI causes a major break down in the potent and highly conserved signalling pathways of leptin and insulin [39,40]. The ATI linked to obesity induces significant local proliferation of pro-inflammatory macrophages compared to anti-inflammatory macrophages within AT. The AT macrophages (ATMs) are produced from bone marrow monocytes. A study demonstrated that *in situ* ATM proliferation is controlled by MCP-1. In the absence of this protein *in vivo*, there was significant reduction ATM in production in genetically obese male mice and their lean control littermates [41].

Most importantly, ATI linked to obesity arising from the chronic accumulation of lipid (trans and saturated fats, low-density lipoprotein - LDL) deposits in AT cells develop from adipocyte extension, deformation, loss of function, and excessive release of pro-inflammatory (IL-1β and TNF-α) compared to anti-inflammatory factors [36]. Studies involving animal models have also provided added evidence on ATI linked to obesity, IR and T2D [12,42]. Xu and Carrero [43] had showed that AT macrophages (ATMs) have a key function in morbid obesity and inflammatory processes leading to chronic inflammatory disease which begins in the AT and is closely associated with IR and comorbidities. Hence, studies surrounding lipid homeostasis *in vivo*, adipocyte cytokines [adiponectin, plasminogen activator inhibitor-1 (PAI-1) regulatory protein, TNF-α, IL-6 etc.] signalling, and ATI related cells and pathways are paramount to drive future research interests and proffer sustainable therapeutic strategies that safeguard health [44].

In addition, in the development of ATI, several inflammation-related transcription factors (TFs) are involved. The mitogen-activated protein kinases (MAPKs, that is, MAPK, MAPKK and MAPKKK tiered cascades) act as key modulators for transduction of extracellular signals to intracellular stimuli [45]. The c-Jun N-terminal kinase (JNK), p38, and extracellular signal-regulated kinase (ERK) are also active members in the MAPK signals pathway, and are key in glucose, adipogenesis and appetite regulation [46]. Once activated, this pathway phosphorylates signals, triggers TFs activation, mediates expression of genes and begins cellular inflammation, propagation, differentiation, and apoptotic events [47,48]. The MAPKs activation induces ATI linked to obesity, and IR upon inactivation of insulin receptor substarte-1 (IRS1) and proliferator-activated receptor (PPAR-γ) genes. Diet or food intake and thermogenesis (BAT) are also positively or negatively affected in the phosphatidylinositol 3-kinase (PI3K)-AKT-mTOR signal pathway. In the Janus kinase-signal transducer and activator of transcription (JAK-STAT) pathway, JAKs stimulate STATs, PI3K and MAPKs while being involved in fat accumulation in the liver and leptin related anorexia [49,50]. Also, it has been reported that the melanocortin (MC) signalling system could have an integral function in obesity development by maintaining energy balance. However, while the mechanism of action remains unclear, the development of anti-obesogenic molecules targeting the MC pathway is still possible [51,52].

## 3 Conventional therapeutic approaches to mitigating ATI linked to obesity

### 3.1 Weight reduction regimens and stimulants

As a medical condition with related comorbidities, obesity development is related to multiple factors which include genetic, humoral, and environmental factors (Fig. 1). Environmental factors include sedentary lifestyle, reduced physical activity, and increased intake of energy dense, high fat or sugar rich foods. In some cases, the possession of specific genes or mutations predisposes an individual to the development of obesity, management becomes more challenging. For example, mutation in the leptin gene or its receptor that regulate hunger and satiety, and mutation in melanocortin-4 receptor (MC-4) or proopiomelanocortin genes may make an individual more prone to obesity [36]. However, gene therapy may provide answers for genetically predisposed obese individuals. On the other hand, weight loss regimens may suffice as a management therapy for environmentally induced obesity. Also, ATI may be mitigated via strict adherence to weight loss regimes that involve dieting and exercise [44].

Unlike obese individuals with larger sized adipocytes, non-obese persons possess smaller adipocytes which enhance metabolic stability. The presence of enlarged adipocytic cells under obese conditions promotes the infiltration of macrophages and ATI development. As such, metabolic dysfunction arises from dysfunctional adipocytes that show inability to function in fat deposition regulation [53]. In a bid to regain metabolic homeostasis, the successful achievement of weight reduction through diet modification (low fat and high fibre diets) with or without exercise have been shown to be efficacious in reducing body mass index (BMI) and obesity [54]. A genetic expression study has shown that the gene expression profile (stromal-vascular AT fraction) of group A (obese subject on low calorie diet) was similar to those of group B (non-obese), compared to the pattern of obese subjects prior to start of low calorie diet [54]. Weight loss therefore achieves improvement in homeostatic cell functions through the reduction of pro-inflammatory factors in obese individuals on low calorie diets. Weight loss benefits on ATI linked to obesity and comorbidities may be due to changes in the inflammatory profile in ATs [55]. A study by Morgan-Bathke and Jensen [56] showed that ATI was reduced in vegans compared to persons on omnivorous diets as indicated by decreased levels of femoral ATMs.

Another study by Colson et al. [57] demonstrated that mouse diet supplementation with polyunsaturated fatty acid (PUFA) led to a significant upregulation of anti-inflammatory factors in AT. However, the study was done under non-inflamed, non-obese conditions [57]. Nevertheless, resolvin lipid mediators derived from n-3 PUFA [docosahexaenoic (DHA) and eicosapentaenoic (EPA) acids] targeted ATI by reducing pro-inflammatory cytokines, macrophage accumulation, and blocking influx of leucocytes into tissues [58]. The PUFAs were also able to prevent excessive adiposity and IR in mice models [59]. Renner et al. [60] also showed increased levels of adiposity (body fat >50 % of body mass) in obese models compared to 22 % in lean mini pigs. The obese mini pig models showed significant adipocyte death and reduced ATI in visceral AT of the diet-induced obese models. This model mimicked the human metabolic syndrome, making it useful in assessing new anti-obesity and related complications therapies.

Weight reduction from increased energy expenditure and caloric restriction lowers adiposity. It also decreases the risk of contracting obesity-associated comorbidities and improve life expectancy [1]. Exercise facilitates the breakdown of carbohydrates and lipids, stabilizes metabolic profile, reduces triglyceride levels, and lowers LDL to high density lipoprotein (HDL) ratio [61]. The mechanism of action behind lipids and triglycerides (TGs) reduction during aerobic exercises is associated with the regulation of serum apolipoprotein C3 (apoC3) levels. The protein reduced TG‐rich lipoproteins and their uptake in the liver by impeding both hepatic and lipoprotein lipase activities [62]. Again, while low apoC3 levels are needed to downregulate lipoprotein-TG profiles, the reverse is the case with higher levels (increased amounts of TGs) *in vivo*. As such, the apoC3 provides a promising target to achieve a hypotriglyceridemic (reduced TGs) state [63]. Under exercise conditions, a study has also shown that in females, hypotriglyceridemia occurs through the improved removal and reduced production of very-low-density lipoprotein (VLDL)-TG, relative to enhanced clearance of VLDL-TG alone, in males. Hence, there is the possibility of developing VLDL-TG and sex-tailored interventions in obesity care [64]. Research has also demonstrated that fat cell particle size is a major predictor of AT-related insulin resistance (ATIR). In contrast, common abdominal ATI biomarker like IL-6, CD14, and CD68, are not closely associated with ATIR [11].

In addition, studies have shown that energy expenditure may also be stimulated by advancing insights into the mechanistic pathways of BrAT. This area of research comes under adipose tissue remodelling. The BrAT has been shown to adaptively respond via stress stimulation of the sympathetic nervous system (SNS). Such stimulation results in activation of immune cells, enhanced sympathetic output to WAT and differentiation in BrATs, and increased number of brown adipocytes which aid energy expenditure in visceral adiposity [65,66]. A study has shown that transplant of 0.1–0.4 g BrAT into mice visceral cavity prevents weight gain. The BrAT transplantation also improved glucose balance in obese mice fed with high calorie diet [67]. In AT remodelling, the AT extracellular matrix (ECM) evolves with hormonal and nutritional stimulants received by adipocytes. The AT ECM include hyaluronan, collagens, and fibronectin [68], and they function as transducers to prevent adipocytic cell breakage. As such, less rigid ECMs are preferred to rigid ones because they allow healthy expansion of AT adipocytes. Rigid ECMs may initiate inflammation, stressed pathways, and visceral deposition of fat [34]. Also, in energy metabolism, the Pparg coactivator 1 alpha's (PGC-1α, a key regulator of genes associated with mitochondrial biogenesis) anti-inflammatory action was shown to be upregulated in muscle cell line (C2C12 myotubes) and decreased the generation of inflammatory cytokine [69]. As such, the increased expression of PGC-1α in white adipose tissues could reduce WATI. This could be an added anti-inflammation mechanism worth targeting [70].

**Fig. 2:**
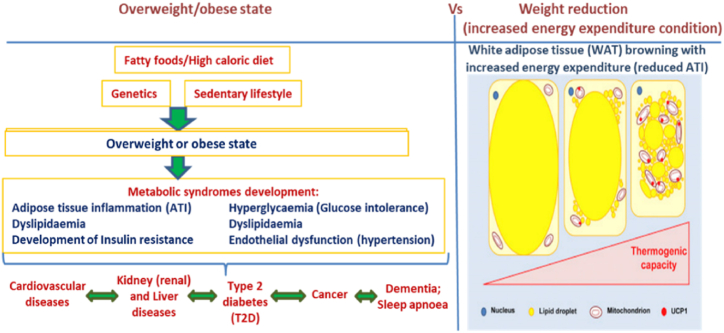
Pathophysiology of obesity in relation to weight reduction or energy expenditure. Ingestion or intake of fatty or high calorie foods result in weight gain, IR and metabolic syndromes development. In contrast, the browning of WAT from up-regulation of their thermogenic capacity (decreased inflammation) lead to weight loss.

Also, fat burning or browning (Fig. 2) through exercise could be a useful therapeutic tool. The WAT browning could be achieved via stimulation of white adipocytes, activation of IL-6, and peroxisome proliferator-activated receptors (PPAR***α*** or PPAR***γ***) inhibition [1]. Furthermore, ***α***-lipoic acid has also been shown to facilitate the brown-like remodelling of cultured subcutaneous white adipocytes from overweight subjects [71]. Again, two new bioactive factors have been proposed in recent years for fat burning. These are musclin secreted by muscles during exercise, and a transcription factor involved in mitochondrial manufacture (TFAM) [72]. The former activates PPAR***γ*** and induces WAT browning (Fig. 2) [72,73]. Irisin is another newly identified adipomyokine involved in WAT browning during energy expenditure [74]. However, irisin's effect on AT differentiation in humans remains a subject of scientific debate [75].

### 3.2 Pharmacotherapies for mitigating ATI linked to obesity

Most pharmaceutical medication regimens target improvements in the systemic inflammation state associated with obesity [44]. Also, a complication-centred approach to the management of ATI linked to obesity have also proved to be successful. Cardio-protective medications with anti-inflammatory activities have also been useful to treat ATI e.g., thiazolidinediones (glitazones) which are PPARγ agonists [44]. They mediate anti-inflammatory effects by controlling the PPARγ target in adipocytes resulting in increased insulin sensitivity. Likewise, a significant reduction in systemic ATI indicators expressed in macrophages and adipocytes is observed. For example, rosiglitazone decreased levels of IL-6 in obese diabetic patients [76]. Pioglitazone reduced MCP-1 levels thereby inhibiting periprostatic WATI [77]. Similar to PPARγ agonists, PPARα agonists like fibrates possess anti-inflammatory and beneficial metabolic effects. Fibrates, angiotensin converting enzyme (ACE) inhibitors, and angiotensin receptor blockers achieve similar reduction in inflammatory mediators such as TNF-α and IL-6. These medications act by mediating activities of inflammatory signal transduction proteins; cyclooxygenase-2 (COX-2) and activator protein-1 [44,78].

Statin drugs also have direct anti-inflammatory activities on adipocytes in ATI linked to obesity. Statin drugs have lipid-lowering potential and act by stalling the key enzyme, 3-hydroxy-3-methlyglutaryl coenzyme A (HMG-CoA reductase). The enzyme functions in cholesterol secretion [5]. Adipocyte cells treated with cerivastatin showed reduced levels of the IL-6 pro-inflammatory factor. The observation suggested that statins could inhibit the prenylation pathway to bring about their anti-inflammatory action [79]. Besides beneficially affecting cytokine expression levels, systemic amounts of serum total adiponectin were also marginally increased in clinical trials involving statins [80]. Simvastatin has also shown attenuation of inflammation in visceral ATs when orally administered to engineered mouse models with diet-induced obesity [5]. The mitigation of ATI by statins correlates with the decrease in inflammatory cell infiltration. Statins reduce MCP-1 secretion levels in obese patients [81]. Studies have also shown that statins have a marked, dose-dependent protective effect on obese patients [82].

Also, COX-2 inhibitors and aspirin have anti-inflammatory activity. However, in terms of cardiovascular contraindications, aspirin was associated with a significant reduction of cardiovascular risk [83], while COX-2 inhibitors raised the risk of myocardial infarction [84]. The differential effect was attributed to platelets. Aspirin stalls the production of platelet-activating thromboxane and inhibits COX-2 and COX-1. On the other hand, COX-2 inhibitor based drugs have vasoactive effects and platelet aggregating properties [84]. While there is little or no evidence to suggest a direct efficacy of COX-2 inhibitors in adipocytes, aspirin may target adipocytes with its anti-inflammatory action [81].

The United States Department of Food and Drug Administration also approved two anti-obesogenic drugs; orlistat (a lipase inhibitor) [85], and lorcaserin [86]. The effect of the former is limited in the long run, while the latter is a new selective serotonin 2C receptor agonist. Lorcaserin stimulates the acuate nucleus pro-opiomelanocortin (POMC) neurons receptor, 5-hydroxytryptamine (5-HT2C), leading to α-MC-stimulating hormone expression. This hormone then acts on paraventricular nucleus MC-4 receptors to increase satisfaction and reduce appetite for food. As such, it has been reported that targeting the 5HT2C receptor may have weight reducing potential [86,87]. Also, anti-diabetic drugs which inhibit dipeptidyl peptidase 4 (DPP4) enzyme activity have been used to stimulate insulin secretion for subsequent blood glucose reduction [88]. Nevertheless, fat loss using the DPP4 inhibitor, evogliptin, has been shown to be better at mediating changes in WAT metabolism and energy expenditure in obese mice than exenatide [89]. The effect of gliptins on body fat mass have not yet been widely investigated in animals and humans, even though various gliptin derivatives have been demonstrated to have a negligible effect on body weight in T2D subjects [90,91]. Koska et al. [92] further showed that saxagliptin may directly reduce ATI, independent of changes in glucose metabolism. Its effect in alleviating ATI was however not significant enough to impact postprandial metabolism. However, a more recent study by Rezki et al. [93] showed a major decrease in postprandial glucose levels in glucose intolerant individuals, following a 12-week saxagliptin therapy regimen which lead to its recommended use in prediabetes care. Saxagliptin acts by preventing the breakdown of glucagon-like peptide 1 GLP-1, reducing glucagon and increasing insulin expression [94]. Moreso, combination of bioactive drugs, for example, metformin and saxagliptin, could lead to improved anti-obesity and anti-T2D outcomes [95]. As such, more effort is required to develop novel anti-obesity pharmaceuticals.

### 3.3 Other potential therapeutic strategies

Certain medical techniques, bioactive platforms, molecules, peptides, and transcription factors possess anti-inflammatory properties, and could be modified or utilized as potential alternative therapies against ATI linked to obesity. Some of them are discussed in this section.

#### 3.3.1 Endogenous erythropoietin (EPO)

This is a glycoprotein hormone associated with erythropoiesis that has demonstrated anti-inflammatory properties, as well as anti-apoptotic activity [96]. The EPO acts as an AT protector and antiapoptotic molecule by reducing the expression of apoptotic molecules like p53, caspases, Bcl 2, Sirt1 and Pp2A. Its action may also be associated with energy metabolism, as well as the insulin [mitogen-activated protein kinase (MAPK), mTOR] and JAK-STAT signal pathways [97]. In a 2015 study, EPO receptor signalling reduced production of inflammatory monocytes and intolerance to glucose, and halted WAT related inflammation (WATI). The treatment of mice on a high-fat diet with EPO significantly reduced pro-inflammatory macrophage-like (M1-like) cells. It also increased anti-inflammatory macrophage-like (M2-like) cells, indicating a reversal in the dynamics linked with obesity-induced ATI [98]. These observations could have positive implications for management of diet-induced obesity and reduce IR associated with WATI. Still, the role played by EPO receptor signalling in adipocyte cells on obesity-induced WATI requires further in-depth research and should not be totally overlooked [98].

#### *3.3.2* Stromal cells secreting interleukin-33 (IL-33)

This an integral factor that regulates adaptive and innate immune homeostasis and immune cell activity in WAT is IL-33. The mechanism of action of IL-33 produced by stromal cells has been linked to inflammatory macrophage regulation in white Ats via the controlled release of IL-4 from eosinophils and IL-5 from type 2 innate lymphoid cytokines (ILC2s) [40,99]. However, the potential sources of IL-33 have remained a subject of scientific debate until recently. Stromal (mesenchymal) cells in WAT were shown to be the dominant producers of IL-33 from adipose stem and progenitor cells (ASPCs), while in visceral WAT mesothelial cells functioned as an additional source. Mesothelial IL-33, upon infection, signals the induction of a peritoneal immune response, while stromal IL-33 facilitates a regulatory cycle. The stromal IL-33 cycle sustains immune balance by maintaining eosinophil levels and inducing innate lymphoid ILC2s. IL-33 role in immune cells’ homeostasis is essential since ATI linked to obesity is associated with the dysfunction of WAT and up-regulation of immune cells [99]. The IL-33 may also be found on other human and animal tissue cell types such as epithelial (lung and intestine), fibroblasts and endothelial cells. IL-33 levels also increase in infectious and inflammatory diseases in humans and mouse models [100].

#### 3.3.3 1,3,6,7-Tetrahydroxy-8-prenylxanthone (TPX)

Recently, TPX which was detected in the hull of the mangosteen fruit (*Garcinia mangostana*) and attenuated ATI. The molecule acts as a potent inhibitor of the IL-6 factor produced in macrophages and adipocytes and alleviates The TNF-α mediated inflammation in adipocytes and inflammatory responses in macrophages by enhancing sirtuin 3 secretion and staling nuclear factor-κB (NF-κB) activation [101].

#### 3.3.4 Gut-axis pathway target

The microbiota of the gut impacts a regulatory effect on obesity associated WATI. Certain metabolites produced by the gut flora which are derive from tryptophan showed control over the expression of the miR-181 family of microRNAs in white adipocytes. Disruption of this gut flora-RNA axis was required for WATI, IR and obesity development. Hence, the miR-181 microRNA family may be a potential target for future therapeutic anti-obesity drug development [102].

#### 3.3.5 Electroacupuncture (EA)

The application of repeated electroacupuncture to obese mice has been demonstrated to reduce hypoxia in AT by lowering the levels of hypoxia-related genes, vascular endothelial growth factor A (VEGFA), and glucose transporter type 1 expression. Likewise, it mitigates ATI through reduction in levels of inflammatory TNF-α, NF-κB, IL-6, MCP-1 expression), macrophage infiltration and body fat mass. The study applied low frequency EA (2 Hz) to male obese mice at the Zusanli (ST36) acupoint area for 10 min every time at a two-day interval for one or two consecutive weeks. However, the effect of EA on obesity and its comorbidities require further studies to identify potential mechanisms for future exploitation in novel therapeutic or preventative strategies [103].

#### 3.3.6 Anti-inflammatory peptides (AIPs) and agents

Reversing or inducing low-level inflammatory responses and modulating immunotolerance remain central to mitigate ATI linked to obesity and achieve homeostasis. The futuristic outlook and search for bioactive peptides (BAPs) with anti-obesogenic efficacy have emerged in light of their potential to be developed as safer alternative therapeutic drugs. Therapeutic peptides can be derived from marine or food sources, and new evolving experimental strategies that are peptidomimetics centred have been used to validate their anti-obesogenic activity [104]. Mimetics, derivatized alternatives, and novel peptide molecules could provide an added source of safe and rarely available anti-obesogenic medications. Peptides such as the GLP-1 used in T2D care, and melanocortin-4 receptor (MC4R)-specific agonists, prolactin-releasing peptide mimetics, neuropeptides, and apolipoprotein A-I have been shown to possess anti-obesity functions [104]. Analogues of bioactive AIPs also exist for new targets and include MetAP2 inhibitors, GLP-1, MC4R, cannabinoid type-1 receptor blockers, oxyntomodulin, lipase inhibitors, leptin, vaccines with anti-obesogenic effect, amylin, and neuropeptide Y antagonists. However, these analogues require more studies even though their combined use and synergistic effects have been predicted to be beneficial [24]. Likewise, molecules like adrenomedullin, cortistatin, ghrelin, melanocyte inducing hormone, urocortin, vasoactive intestinal peptide, all examples of anti-inflammatory hormones (AIHs) and neuropeptides (AINs) have shown beneficial anti-inflammatory effects in laboratory-based models. The AIHs and AINs act via the downregulation of innate immune reactions to achieve T cells synthesis and impede antigen-specific T(H)1-driven reactions [105].

Biologically active peptides (BAPs) have favourable effects *in vivo* against obesity development through the modulation of physiological mechanisms [106,107] like those relating to regulation of satiety, and thus have key roles in the digestive system. The same peptides show cardioprotective functions as they enhance antioxidative responses, inhibit lipid build-up, and prevent hypertension. Peptides are also well tolerated by the immune system particularly when they enhance immune responses [108]. Associated peptides from soybean and rice hydrolysates induced non-specific immune defence and antioxidant effects. The egg white derived AIP, ovotransferrin, was reported to retard the generation of spleen lymphocytes [109]. Egg peptides have also shown α-glucosidase and antidiabetic activities [110]. In terms of metabolic impact, obesity and associated comorbidities like hypertension and T2D arise from metabolic changes linked to low amounts of HDL, and high levels of LDL and triglycerides. However, some therapeutic peptides such as dipeptidyl peptidase IV, α-glucosidase (associated T2D development) function in metabolism regulation [111]. In another study, AIPs such as Val-His, Leu-Ala-Asn, Ala-Leu, Ile-Ala from protein hydrolysates showed good single and synergistic anti-inflammatory activities *in vitro* and could have potential to mitigate inflammation-inducing diseases [112]. Bioactive peptides have also been shown to induce anti-obesity effects and cause body weight reduction in mouse models through the downward regulation of the PPAR-γ pathway [113]. Certain peptides are also able to lower cholesterol levels. Lupin affects the hepatocyte nuclear factor-1 alpha (HNF1α), and sterol regulatory element-binding protein 2 (SREBP2) transcription factors associated with lipid utilization [114]. Also, phenylalanine-proline, a dipeptide, significantly reduces Niemann-Pick C1-Like 1 (NPC1L1) (necessary for cholesterol absorption) microRNA expression [115]. The anti-inflammatory action of BAPs is reported to be linked to JAK-STAT, MAPK, and NF-κB) pathways retardation [113]. Similar mechanisms have been reported for medicinal plant metabolites (capsaicin, turmeric, ginger, etc.) that act singly or in synergy to evince their anti-inflammatory action in obesity and comorbidities care [116].

While BAPs show great promise as potent anti-obesogenic therapeutic agents, their drawback cannot be over looked. They easily undergo hydrolysis, misfolding, are chemical unstable, have short shelf-life and bioavailability, with poor ability to permeate the cellular membrane. In light of these challenges, peptides editing through peptidomimetic technologies could be efficacious in potential drugs development [117]. Another approach could look beyond conventional AIPs to target peptides aptamers, peptide engineering, and multi-functional peptides derivatization to increase the limited number of anti-obesogenic medications. These are alternative, new, and emerging strategies for novel peptides discovery and design [113,118].

#### 3.3.7 Novel integrated platforms

Zhu et al. [119] described an integrated biosensor platform that incorporated AT on a chip for study of ATI linked to obesity at the nano-plasmonic level. The chip could monitor and identify stage-specific cytokine production profiles in a complex mixture during the onset and development of obesity in real time. This suggests that it may have potential use as a high-throughput preclinical readout in personalized obesity care in the near future.

### 3.4 Phytotherapeutics for the mitigation of ATI linked to obesity

#### 3.4.1 Natural phenol compounds and extractable phenol (EP) extracts

Natural phenolic compounds derived from plants such as gallic acid have shown significant anti-obesogenic properties in experimental models. Gallic acid acts by reducing excess storage of lipid and particularly targets the AT to suppress lipogenesis, improve insulin signalling, and down regulate pro-inflammatory chain reactions, as well as oxidative stress in overweight subjects [120]. Glucoraphanin, a compound purified from broccoli sprout extract, also effectively ameliorated ATI linked to obesity. This compound enhanced WAT browning, energy expenditure, reduced insulin resistance and ATI via metabolic endotoxemia reduction and pro-inflammatory macrophages polarization [121]. Protocatechuic acid, an anthocyanin metabolite, has been shown to reinstate insulin sensitivity by stimulating insulin signalling in AT. It also attenuated inflammation in visceral AT in obese subjects [122]. In light of the above, the potential multivariate function of plant-based phenol compounds in future anti-obesogenic therapeutic formulations should not be undermined [123].

Furthermore, phenolic extracts with antioxidant activities may be potentially useful in the management of ATI linked to obesity. The scientific shift to studies on extracts are based on the search for natural and safe therapeutic options. The need for natural formulations is also closely linked to the adverse effects of synthetic anti-obesity medications such heart attacks, insomnia, constipation, nausea, stomach, and headaches [124]. Studies on flavonoid-rich leaf extract of *Cosmos caudatus* showed significant anti-obesity (pancreatic lipase inhibitory effect of 21.7 ± 1.3 %) activity compared to other native Latin American plants (range 10.4 ± 1.0–20.6 ± 2.0 %) at 1000 ppm concentration (Table 1) [125]. Bioactive compounds-rich (polyphenols, tocotrienols and γ-oryzanol) rice bran extract used in diet supplementation over a 20-week period showed ATI markers modulation. Adipocyte size reduced with decreased expression of TNF-α, IL-6, and IL-1β [126]. Similar observations were reported in another study involving Zucker rats [127], as well as for botanical extracts containing quercetin [128], and a Brazilian *Campomanesia phaea* O. Berg fruit extract [129]. The phenol-rich bark extracts of *Prunus yedoensis* [130], *Actinidia polygama* [131], buckwheat [132], and ginseng reduced WAT mass, body weight, mRNA TNF-α, MCP-1, IL-6 and other pro-inflammatory factors in obese mice fed on extract supplemented diet [133].

**Table 1:** EPs and macromolecular antioxidant (MA) compounds and extracts in ATI alleviation.

Plant extract or compound	Phenol profile or active compound(s)	Mechanism of action	Reference(s)
Ethanolic extracts of *Cosmos caudatus*, *Lawsonia inermis*	procyanidin B1, quercetin-3-rhamnoside, catechin, kaempherol, quercetin, quinic acid, 1-caffeyolquinic acid, monogalloyl glucose etc.	Inhibition of lipase activity in fat digestion	[125,134]
*Pluchea indica*, *Carica papaya*, *Piper betle*, *Andrographis paniculata*, *Pereskia bleo* and *Melicope lunu*	Unprofiled polyphenols	Lipase activity inhibition	[125]
Tart cherry and rice bran	Polyphenols, tocotrienols, tocotrienols and g-oryzanol	Reduction of proinflammatory factors, modification of adipocyte size or distribution pattern; diminished secretion of inflammatory adipokinesin 3 T3-L1	[126,127,135]
*Curcuma longa* with white pepper	Curcumin, tetrahydrocurcumin	Decrease expression of inflammatory cytokines in the adipose tissue	[136]
*Campomanesia phaea* O. Berg fruit	Ellagitannins, proanthocyanidins	Downward regulation of pro-inflammatory cytokines; glucose homeostasis	[129]
Red onion and quercetin	Quercetin	Change in adipocyte morphology	[128]
Almond skin	Polyphenols, flavonoids	Facilitates lipolysis in 3T3-L1 adipocytes, inhibition of adipogenesis	[137,138]
Ginseng	Phenols, saponins, ginsenosides	Reduced mRNA levels of adipogenic (PPARγ) and proinflammatory genes (TNF-α, IL-6 and MCP-1)	[133]
*Prunus yedoensis* bark	Unprofiled phenols and flavonoids	Glucose homeostasis; reduced expression of inflammatory and macrophage genes; increase in scavenger receptor genes in AT macrophages	[130]
Pear	Malaxinic acid and other phenols	Reduced adipocyte hypertrophy and WAT inflammation	[139]
*Actinidia polygama,* Bangpoongtongsungsan (BPT)	Unprofiled phenols	Reduction in adipocyte size	[131,140]
Pomegranate husk	Punicalagin, ellagic acid	Reduced production of macrophage, TNF-α, IL-6	[141]
*Hibiscus sabdariffa* L.	Anthocyanins, phenolic acids, flavonoids	Adipogenesis suppression via the PPARγ pathway, inhibition of lipid build-up	[142]
Pomegranate	Ellagitannins	Inhibition of lipase	[143]
Grape	Proanthocyanidins	Lipase activity inhibition	[144]
Walnut	Hydrolyzable tannins, Ellagic, valoneic and dilactone acid	Not yet determined	[145,146]
*Opuntia ficus-indica* cladodes	polyhydroxylated pinellic acid, neohancoside C, polyhydroxypregnane glycoside, isovitexin 7-*O*-xyloside-2″-*O*-glucoside	Not yet determined	[147]

Also, there are clinical studies proving that the consumption of phenol and flavonoid-rich food extracts could lead to beneficial health effects. Flavonoids such as ellagic acid, punicalagin and anthocyanins in pomegranate fruits positively affected the function of adipocytes, as measured in lipogenic and lipolytic activity assays on human AT. The fruit juice staled lipolysis and lipogenesis in human and mouse adipose cells [148], similar to almond skin polyphenol extracts [137]. There was also a major decrease in cholesterol, serum glucose and inflammatory IL-6 levels after oral diet supplementation in overweight subjects for thirty days [149]. Studies on phenol-rich fractions from *Curcuma longa* [136], pomegranate peels [150] and husks [141], showed similar reduced levels in ATI biomarkers, as well as a hypocholesterolaemic effect. The ability of extracts to modulate the gut microflora of obese subjects indicated a prebiotic effect which also enhanced atherogenic markers like LDL-cholesterol. The gut flora may therefore play an essential role in metabolizing plant extract polyphenols for improved absorption and efficacy in diet-induced obese models [150]. A pear extract rich in malaxinic acid has also been described to favourably reverse ATI and showed anti-obesogenic potential in obese mice [139].

An appreciable range of phenol compounds abound in foodstuffs. As such, the recent trend in functional foods development through food product fortification could be explored for enhanced health effects. The use of natural, phenol-rich extracts and phenol compounds could be applicable in obesity and ATI targeted product formulations [151].

#### 3.4.2 Potential impact of macromolecular antioxidant (MA) extracts

Macromolecular antioxidants (MAs) are high molecular weight compounds bound to dietary fibres. The MAs and MA extracts remain emerging fields of research to date [152], and account for more than 50 % of the total polyphenols in plant by-products [153]. Soluble, low molecular weight antioxidants are usually the sole interest in most reports of plant extractable fractions, while MAs are mostly overlooked and are usually less soluble. Overall, non-extractable polyphenols (NEPPs) (hydrolysable tannins, phenolic acids, flavonols) and polymeric non-extractable proanthocyanidins (NEPA) make up the two major classes of MAs. However, MAs are usually used interchangeably with NEPPs [152]. A recent study reported that 64% of phenolic acids in apple peels were present as NEPPs [154]. Several research on novel natural or chemo-synthesized MAs have proven that MAs possess enhanced total phenol, anti-oxidative, and other bioactive properties with promising health benefits [[155], [156], [157]]. The MA extracts may also protect against chronic diseases and metabolic syndrome risk factors such as obesity and its comorbidities through the synergistic action of constituent MAs [148,150,153].

Fruit MAs such as pomegranate ellagitannins [143] and grape proanthocyanidins [144] have been shown to interact with α-glucosidase and lipase enzymes to evince an anti-inflammatory action. The compounds also possess antioxidant activity, and inhibited oxidation of cholesterol and lipids [143,144]. The *Opuntia ficus-indica* nopal MA extracts were also reported to demonstrate stronger radical scavenging action than the EP fractions [147,158]. Novel MAs were also identified from *Opuntia* cladode extracts and included neohancoside C, isovitexin 7-*O*-xyloside-2″-*O*-glucoside, and polyhydroxypregnane glycoside [147]. Within the body, MAs are known to pass through the gut unaltered, but are metabolised and fermented by colonic microflora, and their metabolism produce soluble and absorbable metabolites that provide extended health benefits. In other words, MAs stay in circulation longer than EPs [159]. Released MA metabolites in the colon also reduced lipid oxidation in the colonic mucosa of healthy rats and polyps in mice with colon cancer [160]. The absorbed MAs also showed potential systemic effects in relation to cardio-metabolic risk factors [161,162]. Other health related attributes of MAs include gene expression modulation and anti-proliferative functions [163]. The association of MAs with dietary fibres implies that their joint consumption along with EPs may produce complementary health effects [156,164].

Unfortunately, MAs are mostly unreported in health and nutrition databases because they are not easily expressed in typical aqueous organic plant extractions. However, the total bioactive capacity of a plant or food is a cumulative function of both soluble and MA constituents, especially when foods are consumed whole [156]. Although MA-enhanced nutraceuticals already exist in the international market, more research are still required. Future works could be channelled toward establishing well-grounded knowledge on MA health effects and mechanisms in ATI linked to obesity. Such studies could also include *in vivo* determinations [164]. Lastly, the inclusion of MAs in dietary studies may lead to the acquisition of improved understanding of fibre-bound MAs roles in nutrition and health [156].

### 3.5 Mechanisms of action of extracts and pure phenols in ATI linked to obesity

The mechanisms of action of compounds in plant extracts and isolated phenolic compounds include enhancement of metal ion chelation, mitigation of lipid peroxidation chain reactions, protection of other antioxidant species (β-carotene), coupled antioxidant effect, and maintenance of balanced redox potential through electron (hydrogen) transfer. Phenolic compounds facilitate redox potential difference reduction of other antioxidants, thus enhancing their function individually and synergistically [165]. Plant-derived, natural bioactive polyphenols contain hydroxyl groups which serve for the neutralization of free radical chain reactions. They play a significant role in mitigating disease progression [166]. Oxidative stress (OS) is modulated by polymeric proanthocyanidins via the OS signalling and stress-activated MAPK pathway. The antioxidant action of procyanidin is attributed to its release of hydrogen ion (H^+^) which counteracts free radicals [165,167]. Chlorogenic acid is reported to chelate metal ions (ferrous) and inhibit OS damage by interfering with hydroxyl radical (·OH) generation in the Fenton pathway [168]. The phenolic acid also increases natural antioxidant enzymes (e.g., quinone oxidoreductase 1) secretion and activates nuclear factor erythroid 2–related factor 2 (Nrf2) transcription [169].

The antioxidant and anti-inflammatory activities of plant extracts and purified bioactive phenols are subjects of many research foci since they evince their biological activities through different metabolic pathways [170]. A *Moringa lucida* fraction containing saponins and flavonoids demonstrated good antioxidant action *in vitro*, while reducing pro-inflammatory and upregulating anti-inflammatory factors [170]. In other works, network pharmacology was used to shed light on the molecular mechanism of Kushen (*Sophorae Flavescentis*), a Traditional Chinese Medicine (TCM) for treating inflammation. The study identified inflammation related genes that interacted with Kushen-based flavonoid compounds and kushenol. The Kushen extract regulated inflammation through the IL-6, IL-1β, TNF-α, and the NF-κB signalling pathways [171]. Tannins [172] and other phenol compounds also show anti-inflammatory activity *in vivo* and *in vitro* and modulate inflammatory marker levels such as cyclooxygenase (COX-2) [173,174]. Tannins and flavonoids also possess significant antioxidant action [175]. The up-regulation of anti-inflammatory IL-10, and attenuation of pro-inflammatory mediators linked to lipopolysaccharide (LPS)-induced inflammation have also been reported for plant-based triterpenes (oleanolic, asiatic and maslinic acids) [176,177]. Licorice (*Glycyrrhiza glabra* L.) containing mostly glycyrrhetinic acid, glycyrrhizin and dipotassium glycyrrhizinate also demonstrated antioxidant and anti-inflammatory activities within the gut [178].

Furthermore, beyond being known as potent antioxidants, studies have also shown that curcuminoids, phenolic acids, stilbenes, and flavonoids contribute to genetic expression modulation via epigenetic pathways. Curcumin represses and genistein activates histone acetyltransferase, while fisetin and resveratrol induce histone deacetylase and sirtuins, respectively [[179], [180], [181]]. Phenolic compounds like curcumin and genistein are also associated with regulating the expression of microRNA [182,183]. In addition, the anti-inflammatory activity of phenolic compounds occurs through the inhibition of reactive nitrogen and oxygen species and pro-inflammatory cytokines production [184]. Phenolic metabolites are involved in epigenetic mechanisms and have shown potential in chromatin remodelling and NF-κB activity modulation [185] and thus, are associated with inflammation-prone diseases and immune system modulation [186]. On the other hand, phenolic compounds such as lignin, tannins, anthocyanins are produced using the phenylpropanoid pathway precursors that allow for elaborate structural variation [187]. Phenolics production is governed by transcription factors (regulatory proteins) that modulate the complex secondary metabolites synthesis network. As such they regulate one or more genes involved in the synthetic pathway [188]. The bHLH and MYB proteins are key regulators of transcription in the biosynthesis of phenolic compounds like flavonoids and phenolic acids [189,190]. Other transcriptional factors that regulate genes for phenolics derivation include the GRAS, ERF, WRKY [[191], [192], [193]], and WD40 [194] families. Transcription factors and pathways that govern the production of beneficial anti-obesity phenolic compounds could be engineered to enhance phenols expression and efficacy [195]. Such newly derived phenol-based molecules could constitute novel therapeutic alternatives in ATI and obesity care for the future.

### 3.6 Future prospects

This work provides a first-hand analysis of the potential impact that macromolecular antioxidants (MA), AIPs, other phenols and plant extracts could have in the mitigation of ATI linked to obesity and comorbidities management. The work also shows that more studies elucidating on extracts' mechanism(s) of action are required. Likewise, there is still room for more *in vivo* and human trial experiments to shed more light on both MA and EP extracts’ mechanism(s) of action. In addition, demand still exists for new medical regimens, pharmacological medications, and platforms targeting various pathways, TFs, among others, to restore lipid homeostasis and promote energy expenditure. Likewise, the role of computational studies and machine learning algorithms should not be undermined with respect to the prediction, identification and modelling of novel molecules with anti-inflammatory and anti-obesogenic properties. In the near future a combination of these strategies will make it possible to achieve significant reduction in ATI linked to obesity and comorbidities mortality rates. Furthermore, since regulating satisfaction from diets, lipid storage, release of insulin, energy expenditure and metabolism are key AT attributes, these features may also be considered as targets in various treatment and preventive options. Research could also be aimed at the derivation of molecules targeting signal pathways and TFs associated with inflammatory responses. Also, the fortification of foods with concentrated natural bioactive phenol-rich extracts and compounds to enhance anti-obesogenic effects should not be overlooked. In addition, during ATI, macrophages surrounding adipocytes, increase in adipocyte size, and anti- and proinflammatory expression of adipokines, cytokines, and chemokines constitute major events that occur [196]. However, not much has been done to link sex related factors to these events since research has shown peculiar responses between males and females. Again, since ATI also puts individuals at risk of vascular complications like atherosclerosis [197], elucidation of the ATI-atherosclerotic link, as well as sex-dependent differences in ATI are required in studies with increased sample size to better mitigate ATI and adverse atherosclerotic and metabolic comorbidities associated with obesity. Insights could accelerate efforts to identify new therapeutic targets and develop novel therapeutic and preventative approaches to suit specific needs. Likewise, research into inflammation-obesity links, potential of therapeutic AIPs and their development using peptidomimetic and other technologies are still required to fully comprehend newer underlying mechanisms.

## 4 Conclusion

Understanding the inflammatory mechanisms, pathways and factors that initiate a chain reaction leading to ATI linked to obesity and comorbidities like T2D, cannot be overemphasized. Scientific research has continued to discover new natural molecules and mechanistic pathways aimed at fully comprehending the intricacies associated with ATI linked to obesity. These will help in the development of novel anti-obesogenic and anti-inflammatory therapeutics and preventive strategies. However, given the complex interplay of lifestyle practices (diet), adipocytes, immune cells, and inflammatory responses in the development of the obese state, blocking key pathways and factors surrounding ATI linked to obesity using a range of pharmaceuticals, natural products or holistic strategies could be the preferred route to take.

## Funding statement

The article was supported by the 10.13039/100007431National Research Foundation (NRF ‘Innovation’ Postdoctoral grant number PSTD2204133389, awarded to Dr. C. E. Aruwa), South Africa. This work was also supported by the Directorate of Research and Postgraduate Support, 10.13039/100007648Durban University of Technology (DUT), South Africa.

## Consent for publication

Not applicable.

## Ethics declarations

Not applicable.

## Data availability statement

Data included in article/supp. material/referenced in article.

## CRediT authorship contribution statement

**Christiana Eleojo Aruwa:** Writing – review & editing, Writing – original draft, Resources, Methodology, Investigation, Funding acquisition, Data curation. **Sabiu Saheed:** Writing – review & editing, Project administration, Conceptualization.

## Declaration of competing interest

None.
